# A persistent memory advantage is specific to grapheme-colour synaesthesia

**DOI:** 10.1038/s41598-020-60388-6

**Published:** 2020-02-26

**Authors:** Katrin Lunke, Beat Meier

**Affiliations:** Institute of Psychology, University of Bern, Bern, Switzerland

**Keywords:** Psychology, Human behaviour

## Abstract

For people with synaesthesia ordinary stimuli such as digits or letters induce concurrent experiences such as colours. Synaesthesia is associated with a memory advantage and the aim of this study was to investigate whether this advantage persists across time. We tested recognition memory of four different types of synaesthesia with different inducer-concurrent pairings across two sessions with a one-year retention interval. In the study phase, participants learned three kinds of stimuli (i.e., related to their inducer, related to their concurrent, or synaesthesia-unrelated): music, words and colours. Recognition memory was tested after one hour and after one year. After one hour, grapheme-colour and grapheme-colour-and-sound-colour synaesthetes showed synaesthesia-specific advantages. After one year, only grapheme-colour synaesthetes still showed an advantage. The results imply that a benefit through enhanced colour-processing is particularly strong and that synaesthesia can lead to a long-lasting memory benefit.

## Introduction

For people with synaesthesia, the experience of ordinary stimuli such as digits, letters or words (i.e., inducers) is linked to untypical experiences, for example colours or positions in an imaginary space (i.e., concurrents). There are many different forms of inducer-concurrent associations but so far most studies have focused on grapheme-colour synaesthesia. Results have shown that synaesthesia is associated with cognitive benefits and is characterized by specific neuronal differences^1–4^. In this study, we focus on the memory advantage in synaesthesia. So far, studies have only investigated the impact of synaesthesia on memory across short intervals and mainly for grapheme-colour synaesthesia. It has not yet been tested whether such an advantage persists over time and across different stimulus materials. In the present study, we thus aimed to investigate the longevity of a possible advantage in different types of synaesthesia and for different types of synaesthesia-related and -unrelated stimuli.

Previous studies have found a memory benefit in an ordinary range predominantly for grapheme-colour synaesthesia^5–7^. Rothen, Meier and Ward^8^ reviewed previous studies which tested grapheme-colour synaesthetes and control participants regarding episodic memory performance for verbal and visual stimuli and compared their effect sizes^7,9–11^. There was a consistent memory benefit of grapheme-colour synaesthetes compared to control participants in both recognition and free recall tasks for auditory (Cohen’s *d* = 0.33–1.07) as well as visually presented verbal stimuli (Cohen’s *d* = 0.45–1.84). Moreover, there was a consistent benefit for cued recall of shape-colour and location-colour associations (Cohen’s *d* = 0.31–1.50) as well as for colour-recognition (Cohen’s *d* = 1.09) but not for shape-shape associations (Cohen’s *d* = −0.27) or complex black-and-white figures (Cohen’s *d* = −0.27–0.98), suggesting that performance advantages differ across different stimulus materials. Some of the studies tested whether the performance advantage is due to encoding or retention and suggested a benefit in colour retention^11^.

Bankieris and Aslin^12,13^ investigated whether an advantage in the implicit and explicit encoding of colour-associations is present in grapheme-colour synaesthetes. They found enhanced implicit and explicit encoding and retention in grapheme-colour synaesthetes regarding snowflake-colour associations. These results indicate an advantage in the retention of visual stimuli and especially colour associations for grapheme-colour synaesthetes. The authors concluded that the enhanced ability to form and retain colour associations is the crucial factor in the development of grapheme-colour synaesthesia.

Complementary, Chin and Ward^14^ investigated the subjective strength of and confidence in autobiographic memory in grapheme-colour synaesthesia. They found that synaesthetes showed higher recollection and stronger confidence in autobiographic memories. Moreover, synaesthetes reported more colour details. The authors concluded that synaesthetes attribute more importance to earlier childhood memories and construct more vivid memories which they rehearse more often.

In sum, the literature shows a rather consistent memory advantage for grapheme-colour synaesthesia. Only few studies tested cognitive functions in other types of synaesthesia and these indicate different performance patterns^15,16^. Thus, different mechanisms may underlie different types of synaesthesia.

Meier and Rothen^5^ outlined three possibilities which mechanisms might underlie memory benefits in synaesthesia. First, it is possible that a memory advantage occurs for inducer-related material, such as for graphemes in grapheme-colour synaesthesia. Accordingly, graphemes produce not only a verbal code but also a perceptual (colour) code and thus result in a dual retrieval route which is at the core of the memory benefit. This would be in line with the dual-coding hypothesis^17^. Second, an advantage may occur for concurrent-specific material which might reflect enhanced perceptual processing related to the synaesthetic experience. Third, a more general advantage including synaesthesia-unrelated material may occur due to the opportunities for additional connections in the semantic network offered by the synaesthetic associations^1,3^.

The present project was set up to test these possibilities. It consisted of two sessions separated approximately by one year. This allowed testing potential long-term benefits in different forms of synaesthesia. We recruited four different types of synaesthetes – grapheme-colour-, sound-colour, grapheme-colour-and-sound-colour- and sequence-space synaesthetes regarding a possible recognition memory advantage for inducer-, concurrent-, and synaesthesia unrelated stimuli compared to matched controls. Music and words served as inducer-specific stimuli for grapheme-colour and sound-colour synaesthetes, respectively. Colour stimuli served as concurrent-specific stimuli for grapheme-colour-, sound-colour, and grapheme-colour-and-sound-colour-synaesthetes. For sequence-space synaesthetes, all stimuli were synaesthesia unrelated. Thus, the latter group formed a control group to test the hypothesis of a general memory advantage in synaesthesia which is neither related to the inducer nor the concurrent.

Memory was assessed by the proportion of recognition. Moreover, strength of memory was assessed by remember and know judgements which were used to compute measures of recollection and familiarity^18^.

The results of the immediate test (session 1) showed an advantage for musical stimuli for grapheme-colour-and-sound-colour synaesthetes and an advantage for colours for grapheme-colour synaesthetes^18^. Moreover, the advantage for colour relied on recollection, indicating memory traces to be subjectively stronger. In contrast, the benefit for music relied on familiarity. Both grapheme-colour- as well as sound-colour-synaesthetes showed a benefit for the concurrent(i.e., colours). In contrast, grapheme-colour-and-sound-colour synaesthetes showed rather a benefit for the inducer (i.e., dual coding). Sequence-space synaesthetes did not show any advantage which indicates that there is no general memory advantage across all types of synaesthesia. The results confirmed previous reports that different types of synaesthesia differ in their pattern of cognitive advantages^15,16,18–22^.

The present study presents the results of the second test session to test whether the benefits in recognition memory are stable across a one year retention interval.

## Results

### Memory performance

**Figure 1:**
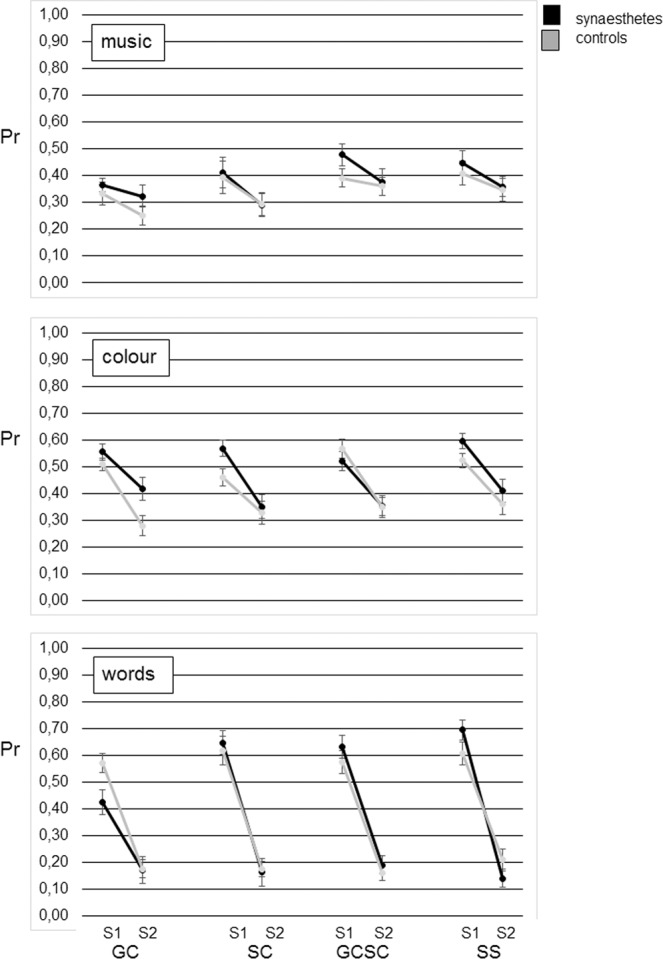
Memory performance (proportion of recognition, Pr) for each *type of synaesthesia* and *each type of* stimuli across a one year interval, separately for synaesthetes (left) and their respective control groups (right), for the different stimulus materials. S1 = Session 1, S2 = Session 2; GC = grapheme-colour synaesthetes, SC = sound-colour synaesthetes, GCSC = grapheme-colour-and-sound-colour synaesthetes, SS = sequence-space-synaesthetes. Error bars represent standard errors.

Figure 1 illustrates memory performance (proportion of recognition, Pr, that is the difference between the proportions of hits and false alarms^23^) in session 1 and session 2 for *each type of synaesthesia*, their group of matched controls, and for each *type of stimuli*.

**Figure 2:**
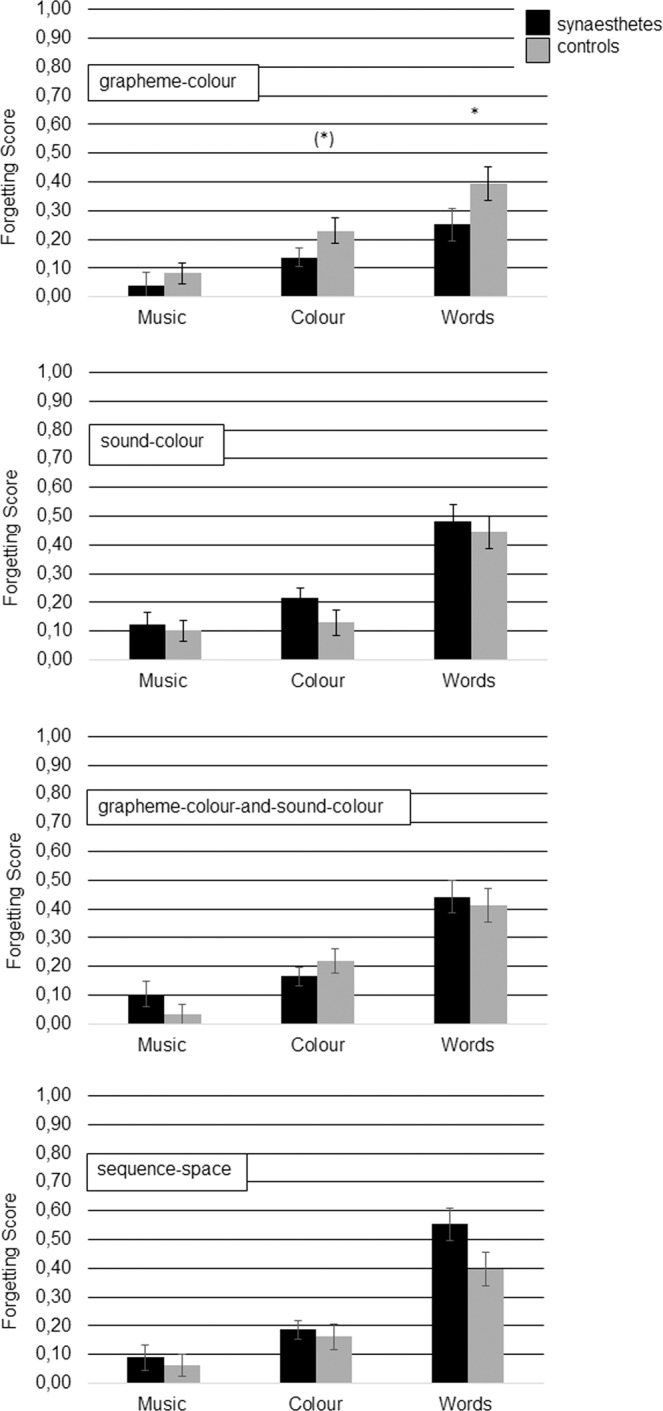
Forgetting scores (difference between proportions of recognition in session 1 and session 2) for each *type of synaesthesia* and each *type of stimuli*. Error bars represent standard errors.

For further analyses, a forgetting score was calculated as difference between memory performance across test sessions (i.e., Pr1 minus Pr2). These scores were analysed separately for each type of synaesthesia. These results are depicted in Fig. 2.

### Forgetting scores

#### Grapheme-colour synaesthesia

For grapheme-colour synaesthesia, a 2 × 3 ANOVA with the between-subject factor *synaesthesia* (yes/no) and the within-subject factor *type of stimuli* (words, colours, music) (Fig. 2) resulted in a main effect of *synaesthesia* (yes/no), *F*(1, 36) = 4.21, *MSE* = 0.06, *p* = 0.048, η_p_^2^ = 0.11, a main effect of *type of stimuli*, *F*(2, 72) = 19.93, *MSE* = 0.03, *p* < 0.001, η_p_^2^ = 0.36 but no interaction, *F*(2, 72) = 0.72, *MSE* = 0.03, *p* = 0.492, η_p_^2^ = 0.02. Planned contrasts were performed as we assumed that grapheme-colour synaesthetes would have an especially small decay for colours. They show that a smaller decay between session 1 and 2 is merely impressive for colours, *t* (36) = −1.68, *p* = 0.051, *d* = −0.55, and words, *t* (36) = −1.74, *p* = 0.045, *d* = −0.57, rather than music, *t* (36) = −0.73, *p* = 0.477, *d* = −0.24. Figure 1 shows however, that for words grapheme-colour synaesthetes started off with a very low performance in session 1 which remained stable while for colours they started off with a high performance and remained high in session 2.

#### Sound-colour synaesthesia

For sound-colour synaesthesia, the same kind of ANOVA gave no main effect of *synaesthesia* (yes/no), *F*(1, 32) = 0.93, *MSE* = 0.07, *p* = 0.342, η_p_^2^ = 0.03, a main effect of *type of stimuli*, *F*(2, 64) = 27.10, *MSE* = 0.04, *p* < 0.001, η_p_^2^ = 0.46 and no interaction, *F*(2, 64) = 0.23, *MSE* = 0.04, *p* = 0.798, η_p_^2^ = 0.01. We conducted Bayesian *t-*tests between synaesthetes and controls for all types of stimuli to determine whether the lack of significant results was due to true null effects, inconclusive data or a lack of power. All results were inconclusive, all *BF*_10_ ≤ 0.70 ≥ 0.34.

#### Grapheme-colour-and-sound-colour synaesthetes

For grapheme-colour-and-sound-colour synaesthesia the same analysis gave no main effect of *synaesthesia* (yes/no), *F*(1, 40) = 0.18, *MSE* = 0.04, *p* = 0.671, η_p_^2^ = 0.01, a main effect of *type of stimuli*, *F*(2, 80) = 31.82, *MSE* = 0.05, *p* < 0.001, η_p_^2^ = 0.44 and no interaction, *F*(2, 80) = 0.96, *MSE* = 0.04, *p* = 0.389, η_p_^2^ = 0.02. Again we conducted Bayesian *t-*tests to determine, whether the lack of significant results was due to true null effects, inconclusive data or a lack of power. For grapheme-colour-and-sound-colour synaesthetes a true null-effect occurred for words (*BF*_10_ = 0.32), but the results were inconclusive for music (*BF*_10_ = 0.58) and colour (*BF*_10_ = 0.39).

#### Sequence-space synaesthetes

For sequence-space synaesthesia, the ANOVA gave no main effect of *synaesthesia* (yes/no), *F*(1, 36) = 2.18, *MSE* = 0.06, *p* = 0.149, η_p_^2^ = 0.06, a main effect of *type of stimuli*, *F*(2, 72) = 45.62, *MSE* = 0.04, *p* < 0.001, η_p_^2^ = 0.56 and no interaction, *F*(2, 72) = 1.52, *MSE* = 0.04, *p* = 0.227, η_p_^2^ = 0.04. Again we conducted Bayesian *t-*tests to determine, whether the lack of significant results was due to true null effects, inconclusive data or a lack of power. There resulted a true null effect for colour, *BF*_10_ = 0.33, results for music, *BF*_10_ = 0.34 and words, *BF*_10_ = 1.62 were inconclusive.

### Recollection and familiarity

Remember/Know judgements were analysed as estimates of recollection and familiarity according to the formula by Yonelinas, Kroll, Dobbins, Lazzara and Knight^24^ (see Statistical Analysis and Design section) Extreme values (e.g. 0) were adjusted according to Stanislaw and Todorov^25^.

We compared whether the subjective strength of memory which had been stronger for grapheme-colour and for sound-colour synaesthetes in session 1^18^ remained stronger in session 2. That is, here we report the most important results (see Supplementary Information for the complete analyses). We conducted four 2 × 3 ANOVAs with the between-subjects factor *synaesthesia* (yes/no) and the within-subject factors *type of stimuli* separately for recollection and familiarity. As in session 1, the analyses revealed that for grapheme-colour synaesthetes and for sound-colour synaesthetes compared to their matched controls significantly higher recollection resulted while familiarity did not differ.

For grapheme-colour-and-sound-colour synaesthetes compared to their matched controls no significant difference materialized neither for recollection nor familiarity. Notably in session 1, familiarity for musical stimuli had been higher^18^.

For sequence-space synaesthetes compared to their matched controls no significant difference materialized neither for recollection nor for familiarity. This is consistent with session 1^18^.

## Discussion

The aim of this study was to test whether a specific memory advantage in synaesthesia persists over time. We tested recognition memory in two sessions that lay 12 months apart. Four types of synaesthesia, grapheme-colour-, sound-colour-, grapheme-colour-and-sound-colour- and sequence-space-synaesthesia, were compared with matched controls on synaesthesia-related and synaesthesia-unrelated stimuli. In session 1, the results had shown a strong advantage for grapheme-colour-and-sound-colour synaesthetes regarding musical stimuli and an advantage for grapheme-colour synaesthetes for colour stimuli. The advantage for music was consistent with dual-coding theory^5,8,17^. Previous studies had shown similar advantages over short delays^10,26^. The advantage for colour in grapheme-colour synaesthetes was consistent with enhanced colour processing. This interpretation is in line with previous studies who found enhanced retention and associative-learning of colours in grapheme-colour synaesthetes^7,11–13^. After one year of retention the advantage of grapheme-colour-and-sound-colour synaesthetes for musical stimuli had vanished while the benefit shown by grapheme-colour synaesthetes for colour stimuli remained significant. Grapheme-colour synaesthetes showed a pattern of generally less forgetting. The discrepancy between two types of grapheme-colour synaesthesia (i.e., pure grapheme-colour synaesthesia and grapheme-colour-and-sound-colour synaesthesia) speaks against a domain-specific advantage.

The advantage for colour shown by grapheme-colour synaesthetes was persistent after one year and thus extends findings by Bankieris and Aslin^12,13^, Pritchard, Rothen, Coolbear and Ward^27^, Rothen and Meier^7^ and Yaro and Ward^11^ who found enhanced memory for colour associations in grapheme-colour synaesthesia after shorter intervals. The results support the hypothesis that the advantage is due to enhanced colour processing. They also support the hypothesis that the advantage is due to a broader semantic network in synaesthesia^1,3^. Presumably grapheme-colour synaesthetes have formed strong episodic bindings between colour stimuli and the situational cues, which is congruent with the explanation that synaesthetes have a broader semantic network with more features to which information can be bound.

The advantage for musical stimuli in grapheme-colour-and-sound-colour synaesthetes did not persist over one year. This result compliments the fact that the advantage for music relied on familiarity in session 1. Recollection based memory is characterized by associations with the encoding situation and localized in episodic memory while familiarity based memory is characterized with a lack of associations with the encoding situation^17^. Memory traces that can be recollected have been encoded with more situational retrieval cues and are thus supposed to be easier to retrieve the next time. Memory traces that are familiarity based are not connected to the encoding situation and thus harder to maintain.

However, this cannot explain why sound-colour synaesthetes did not show any familiarity based advantage for music. In the present study, all types of synaesthesia differed in their pattern of memory advantage even when the inducer-concurrent pairings overlapped. This is, however, consistent with studies by Meier and Rothen^21^ who found even when inducer-concurrent pairings overlapped, different types of synaesthetes showed distinctive cognitive styles. Moreover, it is congruent with a study by Simner *et al*.^15^ who found that sequence-space synaesthetes had synaesthesia-type specific benefits only for some stimuli and tasks. It also complements findings by Ward *et al*.^16^ who found a memory advantage in grapheme-colour but not lexical-gustatory synaesthetes although their inducers overlap. Moreover, Ward *et al*.^22^, Ward, Brown, Sherwood and Simner^28^ and Lunke and Meier^19^ found an impact of the amount of types of synaesthesia present on cognitive functions. Ward *et al*.^22^ found that the more types of synaesthesia present the higher was an advantage in convergent creativity. Ward *et al*.^28^ investigated the impact of the amount of types of synaesthesia onto perception and attention and found a correlation between the amount of types of synaesthesia and the cognitive advantage in perception and attention. Lunke and Meier^19^ found that in different types of synaesthesia only those with multiple types had an advantage in divergent creativity. Types of synaesthesia appear to have different patterns of cognitive advantages with different underlying mechanisms. While grapheme-colour synaesthetes appear to profit from facilitated colour processing and enhanced integration of associations into their semantic network, grapheme-colour-and-sound-colour synaesthetes appear to benefit from dual-coding and broader semantic network *activation*. Familiarity based recognition is related to a fast spreading of activation in the semantic network. Similar mechanism may occur in synaesthetic advantages in creativity. Notably, higher convergent and divergent creativity has been related to fast spreading of activation^29,30^. Thus, familiarity-based activation spreading may be particularly characteristic for multiple synaesthetes and underlie performance in both creativity tasks and some memory tests^19,22^. These mechanisms may work through the number of types of synaesthesia combined, or the inter- or intramodal combination of inducers and concurrents. Future research should investigate whether the differences in underlying mechanisms stem from the comparison between monotypical and multiple synaesthesias or whether it is due to the number of modalities included in inducer-concurrent pairings.

## Conclusion

The present study shows that grapheme-colour synaesthetes had a long-lasting memory advantage, reflected in smaller forgetting, specifically for colour stimuli. In contrast, the immediate memory benefit of grapheme-colour-and-sound-colour synaesthetes for musical stimuli did not last across a one year interval. Notably, neither sound-colour- nor sequence-space synaesthetes showed a memory advantage compared to their matched controls neither immediately nor in terms of forgetting across one year.

The results indicate that different types of synaesthesia show different types of benefit which rely on different mechanisms of memory such as enhanced colour processing for grapheme-colour synaesthesia and probably enhanced colour processing and a broader semantic network. Overall, the study does not support the hypothesis of a general memory advantage for all types of synaesthesia and/or all types of stimuli. Rather, an enduring advantage seems to be restricted to pure grapheme-colour synaesthesia.

## Method

### Participants

76 synaesthetes recruited via the Synaesthesia-Check on the website of the University of Bern (www.synaesthesie.unibe.ch) and 76 healthy control-participants matched for age, gender and education participated^31^. All participants had already participated in session 1 at least 12 months before *M* months = 16.64 (*SD* = 4.50). Eight synaesthetes and six control participants were left handed. Of the synaesthetes, 19 were grapheme-colour synaesthetes (17 female and two male), 17 were sound-colour- (11 female and six male), 21 were grapheme-colour-and-sound-colour (18 female and three male) and 19 sequence-space synaesthetes (all female). Mean age and years of education of the groups of synaesthetes and the respective control groups are summarized in Table 1. Prior to participation in both laboratory sessions, participants filled out an on-line measurement of consistency^32–34^. Mean number of consistent graphemes for grapheme-colour synaesthetes was 25.71 (SD = 6.56) and thus above the cut-off of 20 consistent graphemes, specified by Simner *et al*.^32^ and Rothen and Meier^34^. All participants signed informed consent. The study was approved by the ethics committee of the Faculty of Human Sciences of the University of Bern and all methods were performed in accordance with the relevant guidelines and regulations.

**Table 1:** Mean age and years of education of the groups of synaesthetes and the respective control groups.

		Synaesthetes	Controls
		*M* (*SD*)	*M* (*SD*)
GC	Age	46.79 (18.19)	48.21 (18.03)
	Education	11.53 (1.76)	12.29 (1.81)
SC	Age	35.29 (20.06)	35.53 (21.50)
	Education	12.59 (1.33)	12.94 (1.89)
GCSC	Age	31.33 (11.77)	31.81 (13.40)
	Education	11.74 (1.58)	12.57 (1.33)
SS	Age	32.74 (13.58)	32.16 (14.23)
	Education	12.97 (1.14)	12.61 (1.21)

*Note*. GC = grapheme-colour synaesthetes; SC = sound-colour synaesthetes; GCSC = grapheme-and-sound-colour synaesthetes; SS = sequence-space synaesthetes.

At the beginning of the laboratory session 1, both synaesthetes and control participants were first asked whether they experienced any kind of synaesthesia. If additional synaesthetic experiences were reported to those described before in the Synaesthesia-Check questionnaire, participants were tested for consistency and reassigned. If several types of synaesthesia were present, participants were asked which form they experience as the main type^18,19^.

### Material and apparatus

On-site, participants were tested under controlled light conditions with an 85lux/watt lamp with 6400 calvin colour temperature and two standard interior lamps. Stimuli were presented with E-prime 1.2 (https://www.pstnet.com) on a standard 17 inch flat screen. Answers were given on a standard keyboard and sound was delivered via standard Sennheiser stereo headphones. Audio output was balanced at a comfortable level and remained unchanged during data collection.

#### Word recognition

The word material was composed according to an earlier study^18,35^. The test list for the second session comprised 72 words: 24 words seen twice in session 1, 24 words seen once as distractors in session 1 and 24 unseen new distractor words (originally always 12 high and 12 low frequent words; two words had to be filtered before analysis due to a technical error which resulted in session 2 in 11 high frequency old and new words in condition 1 and 10 high frequency new words in condition 2).

#### Music recognition

Seventy‐two 10 sec wav‐files were recorded from various sources. Each piece of music was selected from unfamiliar, rare recordings. They comprised music styles such as Classic, Jazz, Rock, Pop, Metal, Chinese, Indian, and Swiss folklore. The test list for the second session comprised 24 pieces from the study list of the first session heard twice, 24 from the lures of the first session heard once and 24 unheard new distractors.

#### Colour recognition

Seventy‐two colour patterns were required for colour recognition. Half were selected from a synaesthesia catalogue (International Congress on Synesthesia, Science and Art, Granada, 2009) and half of the patterns were Mondrian style pictures, each consisting of four differently coloured squares. The test list for the second session comprised of 24 patterns from the study list of the first experiment seen twice, 24 patterns seen once as lures in the first experiment and 24 unseen new distractors (always 12 of each of the two categories).

### Procedure

#### Study phase session 1

In the study phase, participants were first presented with a list of words on the screen one at the time. They were instructed to select the colour out of 13 that suited best to each particular word. Colours were retrieved from Simner *et al*.^32^. The words were written in black font on a white background. After a colour was selected the next word appeared. For the music study phase, participants were asked to put on headphones. They were presented with short pieces of music for 10 sec each and were instructed to rate on a seven‐ point scale how much they liked the music. They were also asked whether they knew this particular piece of music. For the colour study phase, participants were presented with coloured patterns for 3 seconds each and they were asked to rate how much they liked each pattern on a seven-point scale. The three study phases were always fulfilled in the same order: words – music – pictures.

#### Recognition phase session 1

In the word recognition test phase, words were presented, one at the time, in randomized order at the center of the screen, in black on a white background. Participants were instructed to indicate whether the word was old or new. After a “new” decision, the next word appeared immediately. After an “old” decision, participants were asked to give a remember/know judgement. To determine subjective strength of memory they were instructed to give a “remember” response when they were able to recollect the word from the study phase and to give a “know” response when they were not able to recollect the word, but nevertheless believed that they had seen it in the study phase. After a response was made, the next word appeared.

In the music recognition test phase, participants were again asked to put on headphones. They were played pieces of music for 10 sec each and they were informed that some of the pieces had been played before (old pieces) and some not (new pieces). They were instructed to indicate whether a piece was old or new. After a “new” decision, the next piece appeared immediately. As in the word recognition test, after an “old” decision, participants were asked to give a remember/know judgement. After a response was made, the next piece was played.

In the colour recognition test, coloured patterns were presented, one at the time, in randomized order at the center of the screen. Participants were informed that some of the patterns were old patterns from the study phase and some were new patterns not presented before. They were instructed to indicate whether a pattern was old or new. After a “new” decision, the next pattern appeared immediately. After an “old” decision, participants were asked to give a remember/know judgement. After a response was made, the next pattern appeared.

#### Session 2

Session 2 was held at least 12 months after session one. The recognition phase of session 2 followed the same procedure as described in session 1: first for word stimuli, then music and colour patterns.

### Statistical analysis and design

To assess recognition for words, music and colours, proportions of hits and false alarms were assessed and the proportions of recognition (Pr) were computed by subtracting proportions of false alarms from proportions of hits. Pr scores were computed for recognition performance in session 1 for stimuli learned and recognized in session 1 (Pr1), recognition performance in session 2 for stimuli learned and recognized in session 1 and recognized again in session 2 (Pr2). For the analyses a difference score *forgetting* was computed as Pr1–Pr2.

Remember/Know judgements were analysed as estimates of recollection and familiarity according to the formula: Recollection = [(Remember old − Remember new)/(1 − Remember new)]; Familiarity = [z(Familiarity old) − z(Familiarity new)], with Familiarity old = [Know old/(1 − Remember old)] and Familiarity new = [Know new/(1 − Remember new)]^24^. Extreme values (e.g. 0) were adjusted by adding 0.5 to these values and 1 to the total with which the proportion was calculated^24^. A detailed analysis of recollection and familiarity is presented in the Supplementary Material while results are shortened in the paper.

With regards to session 1 in which types of synaesthesia differed in their pattern of results and as the sample of session 2 is rather too small for a 2 × 4 × 3 × 2 repeated measures ANCOVA, we decided to analyse the data via four separate ANOVAs for each type of synaesthesia compared with their matched controls. Between-subject factors were *synaesthesia* (yes/no) with two levels and within-subject factor was *type of stimuli* (music, words, colours).

Alpha was set at 0.05 for all analyses. Normality was confirmed with the Kolmogorov-Smirnov test. If homogeneity of variances was violated Greenhouse-Geisser corrected values are reported.

## Supplementary information

Supplementary information

## Data Availability

The datasets generated and analysed during the current study will be available in the BORIS repository, https://boris.unibe.ch.
