# Supplementary material for: A persistent memory advantage is specific to grapheme-colour synaesthesia
Source: Sci Rep. 2020 Feb 26;10:3484. doi: 10.1038/s41598-020-60388-6 (PMC7044321; doi:10.1038/s41598-020-60388-6)
Supplement: Supplementary file 1 — Supplementary information [file 41598_2020_60388_MOESM1_ESM.docx]

Supplementary information

A persistent memory advantage is specific to grapheme-colour synaesthesia

Katrin Lunke & Beat Meier*
University of Bern

Author Note

Katrin Lunke, Beat Meier*, Institute of Psychology, University of Bern.
 This project was supported by the Swiss National Science Foundation, grant 100014_149692
 *Correspondence concerning this article should be addressed to Beat Meier, Insitute of Psychology, University of Bern, Fabrikstrasse 8, 3012 Bern.
E-mail: [beat.meier@psy.unibe.ch](mailto:beat.meier@psy.unibe.ch)

**Analyses of recollection and familiarity**

**Recollection**

*Grapheme-colour-synaesthetes.* We conducted a 2x3 ANOVA with the between-subjects factor synaesthesia (yes/no) and the within-subject factors type of stimuli for recollection (Fig. 1 presents the proportion of remembered and known hits and false alarms from which recollection and familiarity are computed). A main effect occurred for *synaesthesia* (yes/no), *F*(1, 36) = 9.75, *MSE* = 0.02, *p* = .004, η_p_^2^ = .21, no main effect occurred for *type of stimuli, F*(2, 72) = 2.12, *MSE* = 0.02, *p* = .128, η_p_^2^ = .06 and no interaction between *synaesthesia* (yes/no) and *type of stimuli, F*(2, 72) = 1.77, *MSE* = 0.02, *p* = .178, η_p_^2^ = .05.

*Sound-colour synaesthetes.* We conducted a similar 2x3 ANOVA with the between-subjects factor *synaesthesia* (yes/no) and the within-subject factors *type of stimuli* for recollection (Fig. 1). A main effect occurred for *synaesthesia* (yes/no), *F*(1, 32) = 4.17, *MSE* = 0.03, *p* = .049, η_p_^2^ = .12, no main effect occurred for *type of stimuli, F*(1.62, 51.77) = 2.36, *MSE* = 0.01, *p* = .112, η_p_^2^ = .07 and no interaction between *synaesthesia* (yes/no) and *type of stimuli, F*(1.62, 51.77) = 1.12, *MSE* = 0.01, *p* = .324, η_p_^2^ = .03.

*Grapheme-colour-and-sound-colour-synaesthetes.* We conducted a similar 2x3 ANOVA with the between-subjects factor *synaesthesia* (yes/no) and the within-subject factors *type of stimuli* for recollection (Fig. 1). No main effect occurred for *synaesthesia* (yes/no), *F*(1, 40) = 0.00, *MSE* = 0.05, *p* = .992, η_p_^2^ < .01 or for *type of stimuli, F*(2, 80) = 0.61, *MSE* = 0.02, *p* = .547, η_p_^2^ = .02 and no interaction between *synaesthesia* (yes/no) and *type of stimuli, F*(2, 80) = 0.39, *MSE* = 0.02, *p* = .961, η_p_^2^ < .01. We conducted Bayesian *t*-tests between synaesthetes and controls for each type of stimuli to determine, whether the lack of significant results was due to true null effects, inconclusive data or a lack of power. All results were due to true null effects, all *BF*_10_ ≤ 0.31 ≥ 0.30.

*Sequence-space-synaesthetes.* We conducted a similar 2x3 ANOVA with the between-subjects factor *synaesthesia* (yes/no) and the within-subject factors *type of stimuli* for recollection (Fig. 1). No main effect occurred for *synaesthesia* (yes/no), *F*(1, 36) = 0.24, *MSE* = 0.02, *p* = .631, η_p_^2^ = .01. A main effect occurred for *type of stimuli, F*(2, 72) = 5.94, *MSE* = 0.01, *p* = .004, η_p_^2^ = .14 and no interaction between *synaesthesia* (yes/no) and *type of stimuli, F*(2, 72) = 1.54, *MSE* = 0.01, *p* = .222, η_p_^2^ = .04. We conducted Bayesian *t*-tests between synaesthetes and controls for each type of stimuli to determine, whether the lack of significant results was due to true null effects, inconclusive data or a lack of power. For music there resulted a true null effect, *BF*_10_ = 0.32, for colours, *BF*_10_ = 0.42 and words, *BF*_10_ = 1.12 results were inconclusive.

**Familiarity**

*Grapheme-colour-synaesthetes.* We conducted a 2x3 ANOVA with the between-subjects factor *synaesthesia* (yes/no) and the within-subject factors *type of stimuli* for recollection (Fig. 1). No main effect occurred for *synaesthesia* (yes/no), *F*(1, 36) = 0.32, *MSE* = 1.97, *p* = .575, η_p_^2^ = .01 or for *type of stimuli, F*(2, 72) = 1.18, *MSE* = 0.72, *p* = .202, η_p_^2^ = .04 and no interaction between *synaesthesia* (yes/no) and *type of stimuli, F*(2, 72) = 1.24, *MSE* = 0.72, *p* = .295, η_p_^2^ = .03. We conducted Bayesian *t*-tests between synaesthetes and controls for each type of stimuli to determine, whether the lack of significant results was due to true null effects, inconclusive data or a lack of power. All results were inconclusive, all *BF*_10_ ≤ 0.60 ≥ 0.38.

*Sound-colour-synaesthetes.* We conducted a 2x3 ANOVA with the between-subjects factor *synaesthesia* (yes/no) and the within-subject factors *type of stimuli* for recollection (Fig. 1). No main effect occurred for *synaesthesia* (yes/no), *F*(1, 32) = 0.40, *MSE* = 1.40, *p* = .532, η_p_^2^ = .01 or *type of stimuli, F*(2, 64) = 0.05, *MSE* = 0.84, *p* = .592, η_p_^2^ < .01 and no interaction between *synaesthesia* (yes/no) and *type of stimuli, F*(2, 64) = 0.05, *MSE* = 0.84, *p* = .952, η_p_^2^ < .01. We conducted Bayesian *t*-tests between synaesthetes and controls for each type of stimuli to determine, whether the lack of significant results was due to true null effects, inconclusive data or a lack of power. There occurred a true null effect for words, *BF*10 = 0.33, all other results were inconclusive, all *BF*_10_ ≤ 0.42 ≥ 0.35.

*Grapheme-colour-and-sound-colour-synaesthetes.* We conducted a 2x3 ANOVA with the between-subjects factor *synaesthesia* (yes/no) and the within-subject factors *type of stimuli* for recollection (Fig. 1). No main effect occurred for *synaesthesia* (yes/no), *F*(1, 40) = 0.11, *MSE* = 1.40, *p* = .741, η_p_^2^ < .01 or *type of stimuli, F*(1.59, 63.69) = 1.82, *MSE* = 1.09, *p* = .169, η_p_^2^ < .01 and no interaction between *synaesthesia* (yes/no) and *type of stimuli, F*(1.59, 63.69) = 0.09, *MSE* = 1.37, *p* = .8756, η_p_^2^ < .01. We conducted Bayesian *t*-tests between synaesthetes and controls for each type of stimuli to determine, whether the lack of significant results was due to true null effects, inconclusive data or a lack of power. All results were true null effects, all *BF*_10_ ≤ 0.32 ≥ 0.31.

*Sequence-space-synaesthetes.* We conducted a 2x3 ANOVA with the between-subjects factor *synaesthesia* (yes/no) and the within-subject factors *type of stimuli* for recollection (Fig. 1). No main effect occurred for *synaesthesia* (yes/no), *F*(1, 36) = 0.14, *MSE* = 1.29, *p* = .712, η_p_^2^ < .01 or *type of stimuli, F*(2, 72) = 0.54, *MSE* = 0.83, *p* = .586, η_p_^2^ = .02and no interaction between *synaesthesia* (yes/no) and *type of stimuli, F*(2, 72) = 0.84, *MSE* = 0.83, *p* = .435, η_p_^2^ = .02. We conducted Bayesian *t*-tests between synaesthetes and controls for each type of stimuli to determine, whether the lack of significant results was due to true null effects, inconclusive data or a lack of power. All results were inconclusive, all *BF*_10_ ≤ 0.42 ≥ 0.36.


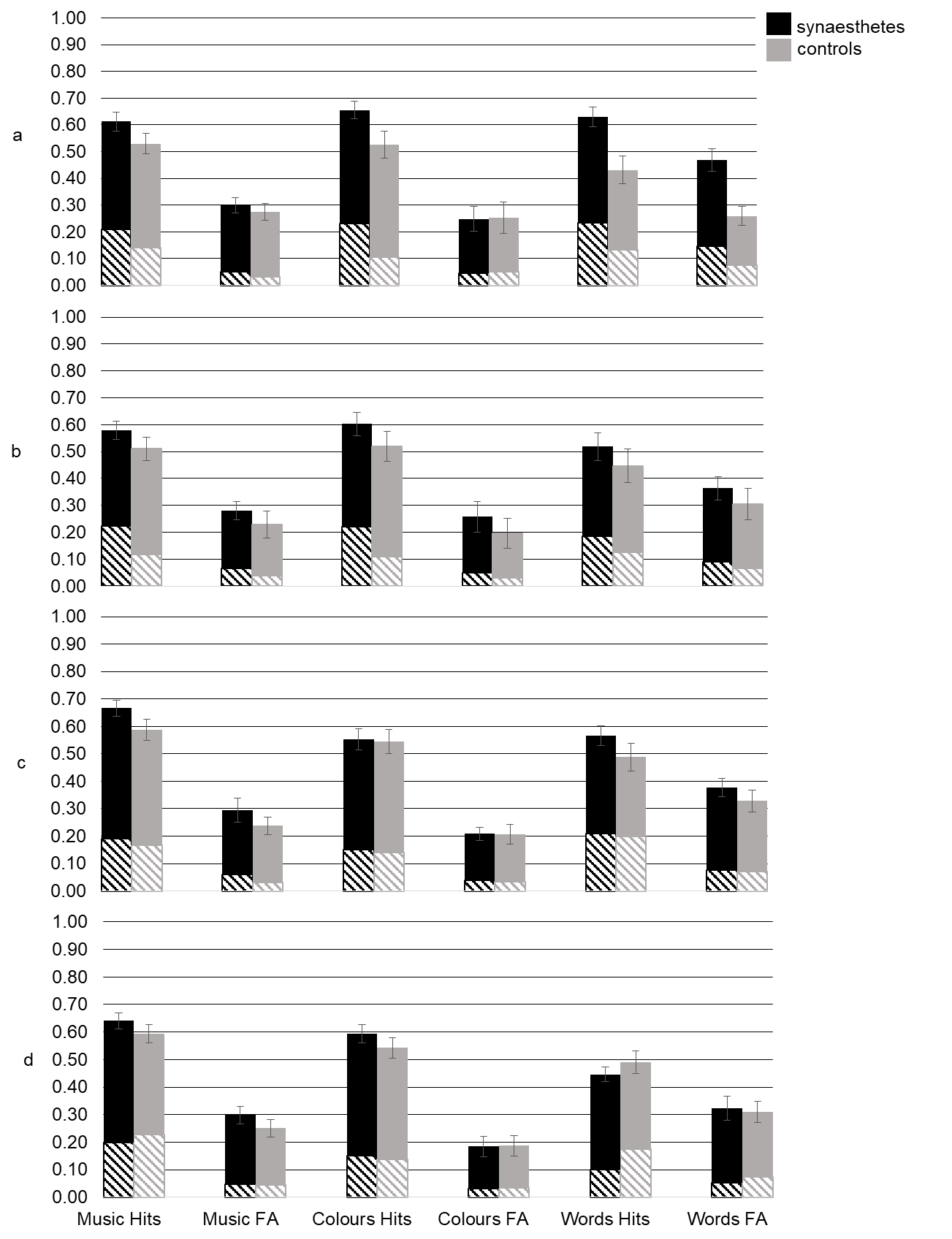


*Figure 1.* Mean proportions of hits and false alarms in session 2 for each *type of synaesthesia* and *each type of stimuli.* The shaded areas reflect *remember*, the solid areas *know* responses. FA = false alarms; a = grapheme-colour synaesthetes, b = sound-colour synaesthetes, c = grapheme-colour-and-sound-colour synaesthetes, d = sequence-space-synaesthetes. Error bars represent standard errors.
